# Gender-specific associations between abdominal adipose mass and bone mineral density in the middle-aged US population

**DOI:** 10.1186/s12891-023-06844-6

**Published:** 2023-09-08

**Authors:** Xueqin Cao, Leilei He, Rong Sun, Siyu Chen

**Affiliations:** 1 Department of Endocrinology, The Dushu Lake Hospital Affiliated to Soochow University, Chongwen Road No. 9, Suzhou, 215000 Jiangsu China; 2 Department of Obstetrics and Gynecology, The Dushu Lake Hospital Affiliated to Soochow University, Chongwen Road No. 9, Suzhou, 215000 Jiangsu China

**Keywords:** Abdominal adipose tissue, Bone mineral density, Osteoporosis, Subcutaneous adipose tissue, Visceral adipose tissue

## Abstract

**Objectives:**

The relationship between abdominal adipose tissue and osteoporosis is poorly understood. The purpose of this study was to examine the associations of abdominal adipose tissue with bone mineral density (BMD) among a nationally representative sample of US middle-aged adults.

**Material and methods:**

This study included 1498 participants from the National Health and Nutrition Examination Survey 2013–2014 and 2017–2018. Dual-energy x-ray absorptiometry was used to measure BMD at the lumbar spine and femoral neck, as well as to assess abdominal adipose mass by categorizing total adipose tissue (TAT) into visceral adipose tissue (VAT) and subcutaneous adipose tissue (SAT). Linear regression was used to assess the relationship between abdominal adipose tissue and BMD, and logistic regression and generalized additive model were used to assess the associations of abdominal adipose tissue with the development of low BMD.

**Results:**

In our study, men accounted for 51.3%, and the mean age and body mass index for men and women were 49.3 and 49.6 years, and 23.9 and 28.3 kg/m^2^, respectively. In the univariate model, we found that abdominal adipose mass was positively associated with BMD at femoral neck and spine in both genders. In the multivariate model, among men, a negative correlation was observed between TAT and SAT and BMD at the femoral neck. Additionally, higher masses of TAT, SAT, and VAT were found to significantly increase the risk of low BMD at both the femoral neck and lumbar spine. In contrast, there was no significant association between abdominal adipose mass and BMD in middle-aged women, regardless of menopausal status.

**Conclusions:**

Our finding suggested that abdominal adipose tissue, regardless of its location (SAT or VAT), may have a negative impact on BMD in middle-aged men independently of body weight, but this relationship was not observed in women. Further research is needed to confirm these findings and investigate potential mechanisms underlying these associations.

**Supplementary Information:**

The online version contains supplementary material available at 10.1186/s12891-023-06844-6.

## Introduction

Osteoporosis is a debilitating disease characterized by low bone mineral density (BMD), microarchitectural deterioration of bone tissue, and an increased risk of fracture [1]. It affects more than 200 million people globally [2], resulting in an estimated 10 million fragility fractures each year [3]. The healthcare and economic burden of osteoporosis on both individuals and society is significant [4]. In the US, approximately 31,000 annual deaths occur within 6 months of hip fracture, with an estimated cost of $17.9 billion on osteoporosis-related fracture annually [5, 6].

The relationship between obesity and osteoporosis is complex and not fully understood [2]. Traditionally, it was believed that obesity, as measured by a high body mass index (BMI), had a protective effect on osteoporosis due to increased mechanical loading on bones [7–10]. However, recent studies have challenged this belief and have shown that deposition of abdominal adipose tissue may have a negative impact on BMD and increase the risk of site-specific fractures [11–13]. Abdominal fat tissue deposition can be broadly categorized into visceral adipose tissue (VAT) and subcutaneous adipose tissue (SAT) [14]. VAT has been strongly linked with multiple cardiovascular risk factors, markers of inflammation and oxidative stress, hepatic steatosis, insulin resistance, and atherosclerosis, and has shown to increase the risk of obesity-related complications, including type 2 diabetes and cardiovascular disease [15–18], while the relationship between SAT and metabolic diseases remains controversial and limited [19–22]. Distribution of adipose tissues seem to be important for bone health, and studies on the relationship between VAT and SAT on bone health have yielded mixed results. While a few studies have reported a positive association between abdominal fat mass, particular VAT, and BMD [23, 24], the majority of studies have reported either no significant relationship or even a negative association after adjusting for BMI [25–29]. Some studies have suggested that the accumulation of SAT presented with greater BMD even after adjustment BMI [19, 20], while others reported that SAT may be detrimental for bone health [21, 22]. The inconsistency of the results may be partly explained by the diversity in study design, sample population and methods applied. Mechanical factors and the propensity of visceral fat to systemic inflammation may also play a role [14, 30, 31]. The interplay between bone and adipose tissue through adipokines, sex hormones, and bone-derived metabolic factors further complicates the relationship, with feedback mechanisms and varied effects on bone remodeling [2, 32]. While osteoporosis is more prevalent in elderly women, bone loss begins to occur in both sexes at age of 40 and continues throughout life [33]. However, to our knowledge, most of the existing studies focused on female and conducted among elderly population.

Therefore, the aim of the present study was to examine the association of abdominal adipose tissue, which was categorized into VAT and SAT, with BMD at femoral neck and lumbar spine in a nationally representative study of middle-aged US men and women.

## Research design and methods

### Study population

The National Health and Nutrition Examination Survey (NHANES), conducted by the National Center for Health Statistics (NCHS), was aimed to evaluate the health and nutritional status of adults and children in the United States. The survey was conducted among a nationally representative sample of approximately 5000 individuals per year, selected from counties across the country, with 15 counties visited annually. Data collection is conducted in accordance with protocols outlined on the NHANES website and with the approval of the institutional review board of the NCHS. All participants provide written informed consent.

In the current study, we analyzed data from the NHANES 2013–2014 and 2017–2018 surveys. Individuals aged from 40 to 59 years with complete available BMD data for the femoral neck and lumbar spine, as well as valid data for TAT, SAT, and VAT were included. Participants with known rheumatoid arthritis, cancer, pregnancy, or missing reasons for menopause and individuals using medications that might influence BMD (e.g., taking prednisone or cortisone daily, and treatment for osteoporosis) were excluded. Finally, and a total of 1498 participants were included in the analysis.

Women were classified as premenopausal if they self-reported not being in menopause and having at least one menstrual period in past 12 months. Women were classified as postmenopausal if they had a surgical history of bilateral oophorectomy, or self-reported being postmenopausal. If menstrual period status was missing, women under 50 years old were classified as premenopausal, and those 50 or older were classified as postmenopausal.

### Assessment of Covariates

Information on race/ethnicity, age, sex, education level, smoking status, physical activity, family income, disease status, and medication use were collected from household interviews questionnaires. Body weight, height and alcohol intake were obtained when people performed health exam in a mobile examination center (MEC). Height was measured using a stadiometer with a fixed vertical backboard and an adjustable head piece, and reported in centimeters. Weight was measured using a digital weight scale and reported in kilogram. BMI was calculated as weight in kilograms divided by height in meters squared. Race/ethnicity was classified as Mexican American, other Hispanic, non-Hispanic white, non-Hispanic black, or other (including multiracial persons). Education level was categorized as less than high school, high school or equivalent, or college or above. Drinking status was grouped into nondrinker, low-to-moderate drinker (defined as < 2 drinks/day in men and < 1 drink/day in women), or heavy drinker (defined as ≥ 2 drinks/day in men and ≥ 1 drink/day in women). Smoking status was classified as never smoker, current smoker, or ever smoker. Leisure activity was categorized into inactive group (no leisure-time physical activity), insufficiently active group (leisure-time moderate activity 1–5 times per week with metabolic equivalents ranging from 3–6 or leisure-time vigorous activity 1–3 times per week with metabolic equivalents > 6), or active group (those who had more leisure-time moderate-or-vigorous activity than above). Family income-to-poverty ratio is an index for the ratio of monthly income to poverty, calculated by dividing family income by the poverty guidelines, specific to family size, as well as the appropriate year and stater [34], and was classified as ≤ 1.0, 1.0–3.0, or > 3.0. Additionally, the total calcium concentration was measured using the Photometric Roche Cobas 6000 Analyzer, and the concentration of total phosphorus was determined utilizing the timed-rate method on a Beckman UniCel® DxC800 Synchron instrument. Rigorous procedures as outlined in the NHANES Laboratory Medical Technologists Procedures Manual were applied throughout blood collection and analysis.

### Body DXA scans

Dual-energy x-ray absorptiometry (DXA) was used to measure participants’ BMD and adipose tissue mass in the MEC during the years 2013–2014 and 2017–2018, and all measurements were performed by well-trained and certified radiology technicians. The scans were acquired on Hologic Discovery model A densitometers (Hologic, Inc., Bedford, Massachusetts), using software version Apex 3.2, with BMD reported in gm/cm^2^ and adipose mass reported in gm. T scores were calculated as $\frac{BMD(target)-mean\;BMD(reference\;group)}{Standard\;deviation(reference\;group)}$, with reference values using BMD data from a young adult reference group in NHANES III [35]. According to World Health Organization (WHO) criteria, osteopenia was defined as − 2.5 < T score < -1.0, osteoporosis was defined as T score ≤ -2.5, and low BMD was defined as a diagnosis of osteopenia or osteoporosis [1]. VAT and SAT mass were measured at the approximate interspace location of L4 and L5 vertebra and body fat percentage (%) was obtained by whole body scans. Details of the DXA examination protocol are documented in the Body Composition Procedures Manual located on the NHANES website [36].

### Statistical analysis

All calculations and statistical analyses for survey data were performed with consideration for strata, cluster, and weight variables to accommodate the sampling scheme. Baseline characteristics were presented as n (weighted percentage) for categorical variables, and weighted mean (95% confidence interval [CI]) for continuous variables. Standard errors were calculated using the Taylor-linearization method. Differences in means or proportions across groups were tested using χ2 test and linear regression model, respectively. We used linear regression models to estimate the regression coefficients (β) and standard errors (SE) for the association of abdominal fat mass with BMD and used logistic models to estimate the odds ratios (ORs) and 95% CI for the association of abdominal fat mass with low BMD risk. The nonlinear relationship of fat mass and BMD was also examined by generalized additive models (GAM). Multivariate model was adjusted for age, race, education, family income-to-poverty ratio, smoking and drinking status, leisure activity, BMI, calcium supplement use, vitamin D supplement use, and diabetes. In addition, for women, hormone replacement therapy and menopause status were also included in the model. Furthermore, we performed stratified analysis by obesity status (defined by BMI and body fat percentage), and menopause status for women. Obesity status was categorized based on the WHO classification guidelines for BMI and body fat percentage. Individuals were classified as underweight if BMI was less than 18.5 kg/m^2^, normal weight if their BMI was between 18.5 to 25.0 kg/m^2^, overweight if their BMI was between 25.0 to 30.0 kg/m^2^, and obese if their BMI was equal to or greater than 30.0 kg/m^2^ [37]. Men with a body fat percentage of 25% or higher and women with a body fat percentage of 35% or higher were considered as obese [38]. All analyses were performed with SAS (Version 9.4, The SAS institute, Cary, NC) and EmpowerStats software (https://www.empowerstats.com). *P* < 0.05 was considered statistically significant.

## Results

Table 1 presents the demographic characteristics of participants. Our study included 1498 participants (768 men and 730 women), with a majority being white (61.5%) and a mean age of 49.5 years. Compared to men, women were less likely to be heavy drinkers or current smokers, more likely to take calcium and vitamin D supplements, and had a higher proportion of osteoporosis (20.2% vs 8.2%) and a lower proportion of diabetes (9.1% vs 13.6%). Among women, 48.3% were postmenopausal and 14.8% were taking hormone replacement therapy. The demographic characteristics of participants according to gender-specific quantiles of TAT, SAT, and VAT mass are presented in Supplementary Table 1–3. With increasing quantiles of TAT mass, participants were less likely to take active leisure physical activities, and more like to have diabetes, however, there was no trend in age. Additionally, compared to those with lower TAT mass, those with higher TAT mass had higher levels of BMI, SAT mass, VAT mass, and BMD at the femoral neck and lumbar spine.

**Table 1:** Characteristics of the participants by sex

	Total	Men	Women	*P*-value
N	1498	768	730	
Race				0.085
Mexican American	234 (9.9)	124 (10.6)	110 (9.2)	
Other Hispanic	153 (6.8)	66 (5.7)	87 (8.0)	
Non-Hispanic White	494 (61.5)	264 (63)	230 (59.7)	
Non-Hispanic Black	293 (11.6)	147 (10.8)	146 (12.5)	
Other Race	324 (10.2)	167 (9.9)	157 (10.5)	
Osteoporosis				< 0.001
Yes	166 (13.9)	56 (8.2)	110 (20.2)	
Education				0.217
Less than high school	301 (14.3)	161 (15.6)	140 (13.0)	
High school or equivalent	325 (24.4)	174 (26.3)	151 (22.3)	
College or above	872 (61.2)	433 (58.1)	439 (64.7)	
Drinking				0.039
Nondrinker	448 (23.8)	189 (20.3)	259 (27.6)	
Low-to-moderate drinker	787 (59.2)	432 (60.6)	355 (57.6)	
Heavy drinker	125 (10.3)	78 (12.4)	47 (8.0)	
Smoking				0.004
Never smoker	917 (60.5)	408 (56.0)	509 (65.4)	
Current smoker	293 (18.5)	182 (21.7)	111 (14.9)	
Ever smoker	288 (21.1)	178 (22.3)	110 (19.7)	
Leisure activity				0.085
No leisure-time physical activity	820 (51.0)	437 (54.8)	383 (46.9)	
Insufficiently active-moderate activity	444 (32.5)	214 (29.4)	230 (35.9)	
Active-moderate activity	233 (16.5)	117 (15.8)	116 (17.2)	
calcium supplement use				0.046
Yes	558 (42.5)	261 (38.5)	297 (46.9)	
vitamin D supplement use				< 0.001
Yes	548 (42.0)	240 (35.7)	308 (48.9)	
Hormone replacement therapy				< 0.001
Yes			83 (14.8)	
Diabetes				0.002
Yes	232 (11.5)	130 (13.6)	102 (9.1)	
Family income-to-poverty ratio				0.564
0–1.0	248 (11)	129 (11.3)	119 (10.7)	
1.0–3.0	471 (27.7)	237 (29.2)	234 (26.0)	
> 3.0	646 (53.7)	329 (52.3)	317 (55.4)	
Menopause				
Yes			328 (48.3)	
Age, years	49.5 (49.1—50.0)	49.3 (48.7—50.0)	49.7 (49.0—50.5)	0.400
BMI, kg/m2	28.7 (28.2—29.1)	29.0 (28.5—29.6)	28.3 (27.7—28.8)	0.048
Total calcium, mmol/L	2.35 (2.34—2.36)	2.35 (2.34—2.36)	2.35 (2.34—2.36)	0.376
Phosphorus, mmol/L	1.20 (1.18—1.21)	1.17 (1.15—1.19)	1.23 (1.21—1.25)	< .001
Subcutaneous adipose tissue mass, kg	1.59 (1.54—1.64)	1.37 (1.31—1.44)	1.82 (1.76—1.89)	< .001
Total abdominal fat tissue mass, kg	2.17 (2.11—2.23)	2.02 (1.94—2.10)	2.33 (2.24—2.41)	< .001
Visceral adipose tissue mass, kg	0.58 (0.56—0.60)	0.65 (0.62—0.67)	0.50 (0.48—0.53)	< 0.001
Femoral neck BMD, gm/cm2	0.81 (0.80—0.82)	0.83 (0.82—0.85)	0.79 (0.78—0.80)	< .001
Lumbar spine BMD, gm/cm2	1.03 (1.02—1.04)	1.04 (1.03—1.05)	1.02 (1.00—1.03)	0.015

Data were presented as n (weighted percentage) for categorical variables and weighted mean (95% CI) for continuous variables

*Abbreviations*: *BMD* Bone mineral density, *BMI* Body mass index, *CI* Confidence interval

The associations of abdominal adipose tissue mass with BMD assessed by linear regressions are shown in Table 2. In univariate analysis, all types of abdominal adipose tissue (TAT, SAT, and VAT mass) were positively correlated with BMD at femoral neck and lumbar spine in both genders. However, after adjusting for other variables in multivariate analysis, negative correlations were observed between abdominal adipose tissue mass and BMD at the femoral neck (STA: β -0.073, *p* = 0.001; TAT: β -0.062, *p* < 0.001; VAT: β -0.086, *p* = 0.054) in men, but not at the lumbar spine. No significant associations were found in women. When further stratified by BMI, negative correlations were observed between TAT and BMD at femoral neck in both non-obese and obese men (β -0.045, *p* = 0.042 and β -0.088, *p* = 0.001, respectively), between TAT and BMD at lumbar spine in obese men (β -0.069, *p* = 0.009), between SAT and BMD at femoral neck in obese men (β -0.099, *p* = 0.006), and between VAT and BMD at femoral neck in non-obese men (β -0.126, *p* = 0.039). Supplementary Table 6 presented the results of stratified analyses conducted by body fat percentage, negatively correlations were observed between VAT and BMD at femoral neck in men and lumbar spine in women (β -0.209, *p* = 0.015 and β -0.195, *p* = 0.010, respectively).

**Table 2:** Relationships between abdominal adipose tissue and bone mineral density assessed by linear regression

	Femoral neck BMD				Lumbar spine BMD			
	Univariate model		Multivariate model		Univariate model		Multivariate model	
	Coefficient (95% CI)	*P* value	Coefficient (95% CI)	*P* value	Coefficient (95% CI)	*P* value	Coefficient (95% CI)	*P* value
TAT								
Men	0.043 (0.027–0.058)	< 0.001	-0.062 (-0.090–0.034)	0.001	0.044 (0.025–0.063)	< 0.001	-0.031 (-0.070–0.008)	0.114
BMI, kg/m^2^								
< 30	0.022 (-0.009–0.052)	0.157	-0.045 (-0.087–0.002)	0.042	0.031 (-0.003–0.065)	0.075	-0.008 (-0.065–0.049)	0.779
≥ 30	0.032 (-0.001–0.065)	0.060	-0.088 (-0.136–0.041)	0.001	0.036 (0.000–0.072)	0.048	-0.069 (-0.119–0.018)	0.009
Women	0.062 (0.049–0.074)	< 0.001	-0.009 (-0.043–0.025)	0.599	0.043 (0.028–0.059)	< 0.001	0.006 (-0.033–0.045)	0.756
BMI, kg/m^2^								
< 30	0.026 (-0.005–0.058)	0.099	0.001 (-0.042–0.044)	0.955	0.006 (-0.033–0.044)	0.761	-0.016 (-0.06–0.027)	0.451
≥ 30	0.038 (0.009–0.067)	0.012	-0.027 (-0.064–0.011)	0.162	0.030 (-0.001–0.060)	0.057	0.011 (-0.048–0.071)	0.701
SAT								
Men	0.066 (0.046–0.087)	< 0.001	-0.073 (-0.111–0.035)	0.001	0.068 (0.044–0.092)	< 0.001	-0.026 (-0.066–0.015)	0.206
BMI, kg/m^2^								
< 30	0.045 (0.001–0.088)	0.044	-0.044 (-0.094–0.006)	0.085	0.056 (0.009–0.103)	0.022	0.004 (-0.074–0.082)	0.911
≥ 30	0.055 (0.010–0.101)	0.019	-0.099 (-0.167–0.030)	0.006	0.063 (0.010–0.116)	0.022	-0.056 (-0.120–0.008)	0.086
Women	0.086 (0.070–0.102)		-0.009 (-0.051–0.034)	0.685	0.063 (0.042–0.083)	< 0.001	0.012 (-0.030–0.054)	0.572
BMI, kg/m^2^								
< 30	0.047 (0.001–0.092)	0.046	0.011 (-0.050–0.072)	0.720	0.025 (-0.025–0.075)	0.317	-0.004 (-0.062–0.055)	0.895
≥ 30	0.059 (0.035–0.084)	< 0.001	-0.040 (-0.081–0.002)	0.059	0.044 (0.015–0.073)	0.004	0.020 (-0.032–0.072)	0.439
VAT								
Men	0.066 (0.009–0.124)	0.025	-0.086 (-0.173–0.002)	0.054	0.070 (0.007–0.134)	0.031	-0.065 (-0.169–0.039)	0.209
BMI, kg/m^2^								
< 30	-0.002 (-0.074–0.07)	0.960	-0.126 (-0.245–0.007)	0.039	0.028 (-0.065–0.122)	0.539	-0.057 (-0.187–0.074)	0.385
≥ 30	0.009 (-0.087–0.106)	0.843	-0.076 (-0.199–0.046)	0.214	0.012 (-0.070–0.094)	0.764	-0.100 (-0.202–0.002)	0.055
Women	0.126 (0.085–0.167)	< 0.001	-0.012 (-0.075–0.051)	0.701	0.075 (0.028–0.122)	0.003	-0.007 (-0.095–0.081)	0.872
BMI, kg/m^2^								
< 30	0.011 (-0.063–0.086)	0.759	-0.032 (-0.13–0.066)	0.511	-0.068 (-0.177–0.041)	0.211	-0.079 (-0.194–0.037)	0.176
≥ 30	-0.026 (-0.119–0.066)	0.564	0.010 (-0.078–0.098)	0.819	-0.013 (-0.102–0.076)	0.773	-0.011 (-0.106–0.084)	0.811

Linear regression models were used to estimate the regression coefficients and 95% (CI) for the association of fat tissue with BMD. Multivariate model was adjusted for age, race, education, family income-to-poverty ratio, smoking and drinking status, leisure activity, body mass index, calcium supplement use, vitamin D supplement use, and diabetes, and for women, hormone replacement therapy, and menopause status

*Abbreviations*: *CI* Confidence interval, *SAT* Subcutaneous adipose tissue, *TAT* Total adipose tissue, *VAT* Visceral adipose tissue

**Fig. 1:**
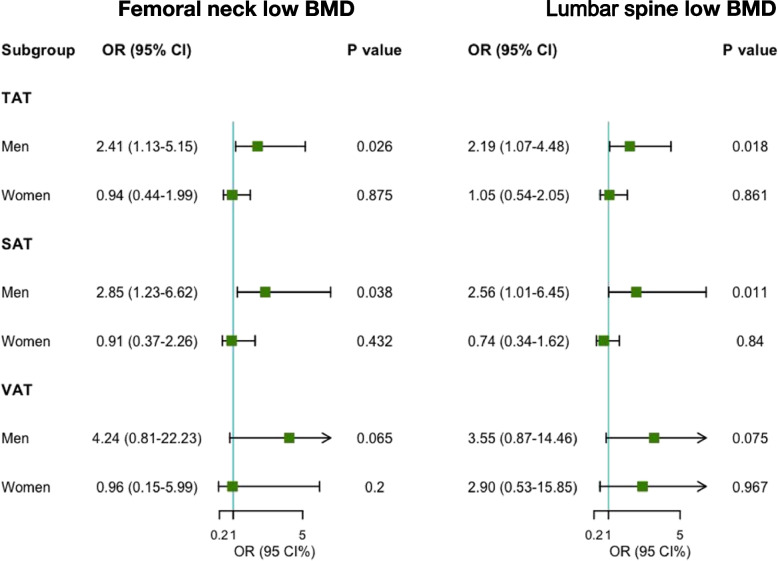
Title: Associations of abdominal adipose tissue with the risk of low BMD assessed by logistic regression. Legend: Logistic model was used and adjusted for age, race, education, family income-to-poverty ratio, smoking and drinking status, leisure activity, body mass index, calcium supplement use, vitamin D supplement use, and diabetes, and for women, hormone replacement therapy, and menopause status

**Fig. 2:**
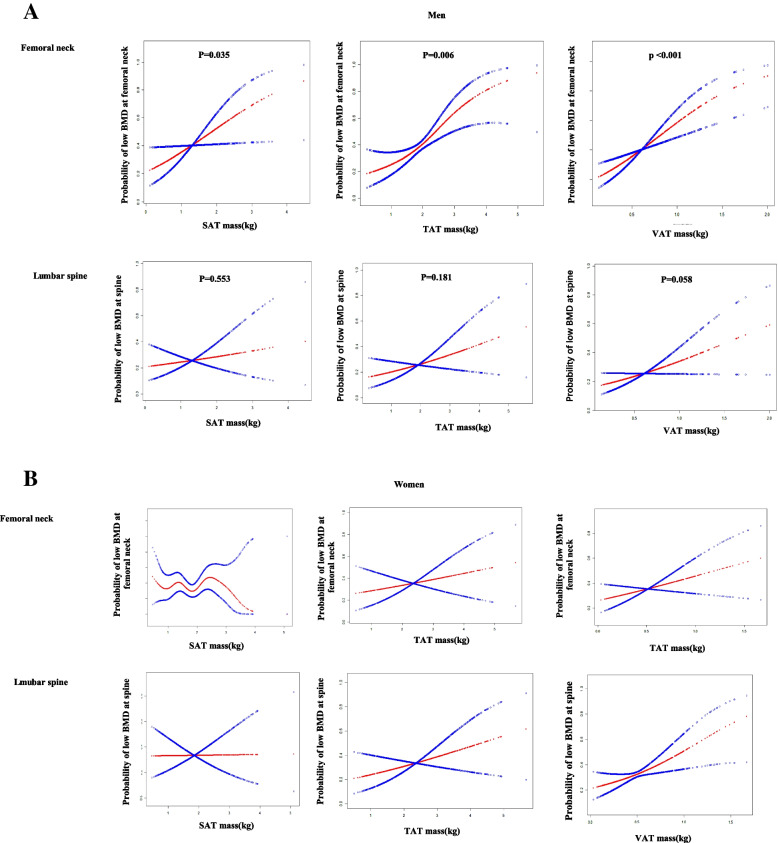
**A** Title: Associations of abdominal adipose tissue with the risk of low BMD assessed by a GAM in men. Legend: A weighted generalized additive model and a smooth curve fitting were performed to assess the relationship between SAT, TAT, VAT and risk of low BMD at femoral neck and spine in men. Blue lines represent 95% confidence intervals. Covariates, including age, race, education, family income-to-poverty ratio, smoking and drinking status, leisure activity, body mass index, calcium supplement use, vitamin D supplement use, and diabetes were adjusted in the models. **B** Title: Associations of abdominal adipose tissue with the risk of low BMD assessed by a generalized additive model in women. Legend: A weighted generalized additive model (GAM) and a smooth curve fitting were performed to assess the relationship between SAT, TAT, VAT and risk of low BMD at femoral neck and spine in women. Blue lines represent 95% confidence intervals. Covariates, including age, race, education, family income-to-poverty ratio, smoking and drinking status, leisure activity, body mass index, calcium supplement use, vitamin D supplement use, and diabetes, and for women, hormone replacement therapy, and menopause status, were adjusted in the models. All *P* value > 0.05

As presented in Fig. 1, multivariable logistic regression analysis was used to assess the associations of abdominal adipose tissue with the risk of low BMD (osteopenia/osteoporosis, T score < -1.0). In men, a kilogram increase in SAT mass was associated with a 2.85 times higher risk of low BMD at femoral neck and a 2.56 times higher risk of low BMD at lumbar spine. Similarly, a kilogram increase in TAT mass was linked to a 2.41 times higher risk of low BMD at femoral neck and a 2.19 times higher risk of low BMD at lumbar spine. These associations were not found to be significant in women. Additionally, using the generalized additive model, nonlinear relationships between abdominal adipose tissue (TAT, SAT, and VAT mass) and risk of low BMD at femoral neck were observed in men, but not in women (Fig. 2A and B).

Stratified analyses were also conducted by menopausal status (non-menopause, menopause) in women. Our results showed that in women, the correlation between abdominal adipose tissue and BMD at the femoral neck and lumbar spine did not reach statistical significance, regardless of menopausal status (See Supplementary Tables 4 and 5).

## Discussion

The current study is the largest study to extensively evaluate the association of abdominal adipose tissue with BMD in the middle-aged adults. Our findings revealed that increased abdominal adipose tissue mass, including TAT, SAT, and VAT, was significantly inversely correlated with BMD at the femoral neck, and increased the risk of low BMD at the femoral neck and lumbar spine in middle-aged men. However, this association was not observed in women, regardless of menopausal status.

Obesity, as measured by BMI, has been traditionally thought to have a positive association with increased BMD [23, 39, 40]. This is supported by the "mechanostat theory" of bone remodeling, which states that bones adapt to mechanical loading by increasing their density, further reinforcing the idea that obesity may provide protection against osteoporosis [41]. A previous study in the same population as ours found that android and gynoid fat mass was positively associated with BMD [23], with android fat predominantly located around the trunk and upper body, and gynoid fat concentrated in areas such as the buttocks, hips and thighs [42, 43]. However, since the previous study did not incorporate BMI into its considerations, it can’t be definitively concluded whether mechanical loading or the very nature of fat mass itself had the definitive role in influencing BMD.

Our present study focused on the abdominal adipose tissue which classified into SAT and VAT. VAT refers to the fat stored in and around abdominal viscera in mesentery and omentum, while SAT is the fat stored beneath the skin in the subcutaneous tissue [14, 31]. Recent evidence suggested that excessive abdominal fat, specifically visceral fat, may have a negative impact on bone health [44–47]. Epidemiological studies have shown that simple abdominal obesity indexes, like higher waist circumference or higher waist to hip ratio, and abdominal obesity as measured by gold standards such as DXA or CT, were associated with lower BMD or higher fracture risk [29, 48–51]. Additionally, recent accumulating evidences suggested that visceral adiposity might be deleterious to bone microarchitecture [20, 48, 49], and was associated with lower BMD and increased osteoporotic vertebral compression refractures [29, 52–54]. Evidence regarding the relationship between SAT and BMD is still limited and controversial. Some studies reported a positive association, indicating that subcutaneous fat beneficially impacted bone structure and strength in healthy individuals [20]. This could, in part, be due to the hormone leptin, produced by subcutaneous fat, may increase bone mass by stimulating osteoblast activity [20, 55, 56]. However, these findings mainly come from studies conducted with nonobese or slightly obese individuals [56–58]. Study conducted among postmenopausal females or obese women found that subcutaneous fat was negatively associated with BMD [59], while others reported no association [60, 61].

In the present study, we found negative correlations between abdominal adipose tissue, including TAT, SAT, and VAT, and BMD at the femoral neck in men, but not in women. In addition, every kilogram of increase in SAT or TAT mass was associated with a 2.19 to 2.85 times higher risk of low BMD at the femoral neck or lumbar spine in men. Analysis using GAM also showed a non-linear relationship between VAT and the risk of low BMD at the femoral neck in men. It was reported that increase in adipocytes might cause a decrease in osteoblasts, and dysfunctional abdominal adipose may release pro-inflammatory cytokines that inhibit bone formation and increase bone resorption, leading to an imbalance in the bone remodeling process [53, 62–64]. Consistent with our study, other studies also showed a gender-specific differences in the association between fat mass and BMD [65–67]. For instance, a study suggested that fat mass was negatively associated with BMD in men but not in women [65]. Additionally, other studies have also shown that fat mass had a stronger negative effect on BMD in men compared to women [66, 67]. The potential mechanisms remain unclear. It is worth noting that men and women differ in the patterns of fat deposition, fat mobilization, and the consequences of both excess and insufficient fat stores [42, 68]. Women are more likely to deposit fat subcutaneously and on their lower extremities; men are more likely to deposit fat in the abdominal region. This ‘female’ fat distribution, independent of total body fat, confers protection against metabolic diseases, such as type 2 diabetes and atherosclerosis, which partly owing to the role of sex hormones, as well as the microenvironment and cell-specific properties within fat depots [68–70]. However, more research is needed to understand the impact of gender and visceral adiposity on bone health.

Our BMI stratified analysis revealed that even in non-obese men, increased mass of abdominal adipose tissue, including TAT and VAT, were significantly associated with lower BMD at the femoral neck. Similarly, in obese men, increased mass of abdominal adipose tissue, including TAT and SAT, were significantly associated with lower BMD at the femoral neck. These findings suggest that abdominal fat may have a negative impact on bone health, particularly at the femoral neck, in men regardless of overall body weight. We found that there is still a negative association between VAT and BMD among individuals with lower body fat percentage when stratified analysis was conducted by body fat percentage, indicating that VAT may have an independent impact on bone health, beyond its correlation with overall body fat percentage. In addition, we found that the relationship between abdominal adipose mass and BMD was non-significant for women, regardless of menopausal status. Although it is well-established that menopause is associated with changes in abdominal fat distribution and increases in visceral fat, which are linked to circulating estrogen levels [71–73], our findings suggested that these changes may not have a significant impact on bone health in women. Estrogen plays a key role in maintaining bone health, and a decrease in estrogen levels during menopause is a major risk factor for osteoporosis [74]. Abdominal fat is a source of estrogen, which may partially compensate for the drop in estrogen levels during menopause [75]. However, evidence regarding the effect of menopause on the association of fat mass and BMD is limited. A previous study suggested that menopause status has a negligible effect on the association between abdominal fat and metabolic syndrome components in overweight women, indicating that other factors may play a more important role in determining bone health in women [76]. Nonetheless, further research is needed to fully understand the relationship between menopause-related changes in abdominal fat and bone health.

Our study had several strengths, including the use of a nationally representative sample of US adults, which enhances the generalizability of the results. Additionally, we adjusted for multiple covariates, including demographic, lifestyle, and health factors, which helped to control for potential confounding variables. We also used DXA, a widely accepted method for measuring bone density and body composition. However, there were also some limitations to our study. First, our study had a limited sample size, and further research with larger samples is necessary to confirm our findings. Second, our study did not measure serum biomarkers of inflammation which play a key role in potential mechanisms. Third, as this study was a cross-sectional observational study, it cannot establish causality, and more prospective research is needed to explore the association between abdominal adipose tissue and BMD.

## Conclusions

In conclusion, in this US population-based study, we found that abdominal adipose mass (TAT, SAT, VAT) was significantly negative associated with BMD at femoral neck, and increased risk of low BMD of femoral neck and lumbar spine in middle-aged men. The relationship between abdominal adipose mass with BMD at both femoral neck and lumbar spine was non-significant in women, independently of menopausal status.

### Supplementary Information

**Additional file 1: Supplementary Table 1.** Characteristics of the participants by total abdominal fat tissue mass and sex. **Supplementary Table 2.** Characteristics of the participants by subcutaneous adipose tissue mass and sex. **Supplementary Table 3.** Characteristics of the participants by visceral adipose tissue mass and sex. **Supplementary Table 4.** Relationships between abdominal adipose tissue and BMD stratified by menopausal status assessed by linear regression. **Supplementary Table 5.** Relationships between abdominal adipose tissue and the risk of low BMD stratified by menopausal status assessed by logistic regression. **Supplementary Table 6. **Relationships between abdominal adipose tissue and BMD stratified by obesity statuses defined by body fat percentage assessed by linear regression.

## Data Availability

The survey data are publicly available on the Internet for data users and researchers throughout the world, for detailed information, see the NHANES website: http://www.cdc.gov/nchs/nhanes.

## Abbreviations

BMD: Bone mineral density
BMI: Body mass index
CI: Confidence interval
DXA: Dual-energy x-ray absorptiometry
GAM: Generalized additive models
NHANES: National Health and Nutrition Examination Survey
NCHS: National Center for Health Statistics
OR: Odds ratio
SAT: Subcutaneous adipose tissue
SE: Standard errors
TAT: Total adipose tissue
VAT: Visceral adipose tissue
β: Regression coefficients

## References

[1] Kanis JA, Melton LJ, Christiansen C, Johnston CC, Khaltaev N (1994). The diagnosis of osteoporosis. J Bone Miner Res.

[2] Gkastaris K, Goulis DG, Potoupnis M, Anastasilakis AD, Kapetanos G (2020). Obesity, osteoporosis and bone metabolism. J Musculoskelet Neuronal Interact.

[3] Hernlund E, Svedbom A, Ivergard M, Compston J, Cooper C, Stenmark J (2013). Osteoporosis in the European Union: medical management, epidemiology and economic burden. A report prepared in collaboration with the International Osteoporosis Foundation (IOF) and the European Federation of Pharmaceutical Industry Associations (EFPIA). Arch Osteoporos.

[4] Tarrant SM, Balogh ZJ (2020). The global burden of surgical management of osteoporotic fractures. World J Surg.

[5] Bone Health and Osteoporosis (2004). A Report of the Surgeon General.

[6] Clynes MA, Harvey NC, Curtis EM, Fuggle NR, Dennison EM, Cooper C (2020). The epidemiology of osteoporosis. Br Med Bull.

[7] De Laet C, Kanis JA, Oden A, Johanson H, Johnell O, Delmas P (2005). Body mass index as a predictor of fracture risk: a meta-analysis. Osteoporos Int.

[8] Looker AC, Flegal KM, Melton LJ (2007). Impact of increased overweight on the projected prevalence of osteoporosis in older women. Osteoporos Int.

[9] Compston JE, Watts NB, Chapurlat R, Cooper C, Boonen S, Greenspan S (2011). Obesity is not protective against fracture in postmenopausal women: GLOW. Am J Med.

[10] Felson DT, Zhang Y, Hannan MT, Anderson JJ (1993). Effects of weight and body mass index on bone mineral density in men and women: the Framingham study. J Bone Miner Res.

[11] Johansson H, Kanis JA, Oden A, McCloskey E, Chapurlat RD, Christiansen C (2014). A meta-analysis of the association of fracture risk and body mass index in women. J Bone Miner Res.

[12] Yoo HJ, Park MS, Yang SJ, Kim TN, Lim KI, Kang HJ (2012). The differential relationship between fat mass and bone mineral density by gender and menopausal status. J Bone Miner Metab.

[13] Svejme O, Ahlborg HG, Nilsson JA, Karlsson MK (2012). Early menopause and risk of osteoporosis, fracture and mortality: a 34-year prospective observational study in 390 women. BJOG.

[14] Tchkonia T, Thomou T, Zhu Y, Karagiannides I, Pothoulakis C, Jensen MD (2013). Mechanisms and metabolic implications of regional differences among fat depots. Cell Metab.

[15] Nicklas BJ, Penninx BW, Cesari M, Kritchevsky SB, Newman AB, Kanaya AM (2004). Association of visceral adipose tissue with incident myocardial infarction in older men and women: the Health, Aging and Body Composition Study. Am J Epidemiol.

[16] Patel P, Abate N (2013). Body fat distribution and insulin resistance. Nutrients.

[17] Matsuzawa Y, Shimomura I, Nakamura T, Keno Y, Tokunaga K (1995). Pathophysiology and pathogenesis of visceral fat obesity. Ann N Y Acad Sci.

[18] Fang H, Berg E, Cheng X, Shen W (2018). How to best assess abdominal obesity. Curr Opin Clin Nutr Metab Care.

[19] Deere K, Sayers A, Viljakainen HT, Lawlor DA, Sattar N, Kemp JP (2013). Distinct relationships of intramuscular and subcutaneous fat with cortical bone: findings from a cross-sectional study of young adult males and females. J Clin Endocrinol Metab.

[20] Gilsanz V, Chalfant J, Mo AO, Lee DC, Dorey FJ, Mittelman SD (2009). Reciprocal relations of subcutaneous and visceral fat to bone structure and strength. J Clin Endocrinol Metab.

[21] Crivelli M, Chain A, da Silva ITF, Waked AM, Bezerra FF (2021). Association of visceral and subcutaneous fat mass with bone density and vertebral fractures in women with severe obesity. J Clin Densitom.

[22] Bland VL, Klimentidis YC, Bea JW, Roe DJ, Funk JL, Going SB (2022). Cross-sectional associations between adipose tissue depots and areal bone mineral density in the UK Biobank imaging study. Osteoporos Int.

[23] Ma M, Liu X, Jia G, Geng B, Xia Y (2022). The association between body fat distribution and bone mineral density: evidence from the US population. BMC Endocr Disord.

[24] Li M, Lv F, Zhang Z, Deng W, Li Y, Deng Z (2016). Establishment of a normal reference value of parathyroid hormone in a large healthy Chinese population and evaluation of its relation to bone turnover and bone mineral density. Osteoporos Int.

[25] Sharma DK, Anderson PH, Morris HA, Clifton PM (2020). Visceral fat is a negative determinant of bone health in obese postmenopausal women. Int J Environ Res Public Health.

[26] Liu CT, Broe KE, Zhou Y, Boyd SK, Cupples LA, Hannan MT (2017). Visceral adipose tissue is associated with bone microarchitecture in the framingham osteoporosis study. J Bone Miner Res.

[27] Luo J, Lee RY (2020). How does obesity influence the risk of vertebral fracture? findings from the UK biobank participants. JBMR Plus.

[28] Zhu K, Hunter M, James A, Lim EM, Cooke BR, Walsh JP (2020). Relationship between visceral adipose tissue and bone mineral density in Australian baby boomers. Osteoporos Int.

[29] Zhang P, Peterson M, Su GL, Wang SC (2015). Visceral adiposity is negatively associated with bone density and muscle attenuation. Am J Clin Nutr.

[30] Lumeng CN, Bodzin JL, Saltiel AR (2007). Obesity induces a phenotypic switch in adipose tissue macrophage polarization. J Clin Invest.

[31] Ibrahim MM (2010). Subcutaneous and visceral adipose tissue: structural and functional differences. Obes Rev.

[32] Gonnelli S, Caffarelli C, Nuti R (2014). Obesity and fracture risk. Clin Cases Miner Bone Metab.

[33] Khosla S, Riggs BL (2005). Pathophysiology of age-related bone loss and osteoporosis. Endocrinol Metab Clin North Am.

[34] NHANES 2018. Available from: https://wwwn.cdc.gov/Nchs/Nhanes/2017-2018/INQ_J.htm.

[35] Looker AC, Orwoll ES, Johnston CC, Lindsay RL, Wahner HW, Dunn WL (1997). Prevalence of low femoral bone density in older U.S. adults from NHANES III. J Bone Miner Res.

[36] NHANES. Body_Composition_Procedures_Manual 2018. Available from: https://wwwn.cdc.gov/nchs/data/nhanes/2017-2018/manuals/Body_Composition_Procedures_Manual_2018.pdf.

[37] Physical status: the use and interpretation of anthropometry (1995). Report of a WHO Expert Committee. World Health Organ Tech Rep Ser.

[38] Obesity: preventing and managing the global epidemic (2000). Report of a WHO consultation. World Health Organ Tech Rep Ser.

[39] Tan CY, Vidal-Puig A (2008). Adipose tissue expandability: the metabolic problems of obesity may arise from the inability to become more obese. Biochem Soc Trans.

[40] Bays HE, Gonzalez-Campoy JM, Bray GA, Kitabchi AE, Bergman DA, Schorr AB (2008). Pathogenic potential of adipose tissue and metabolic consequences of adipocyte hypertrophy and increased visceral adiposity. Expert Rev Cardiovasc Ther.

[41] Kim CJ, Oh KW, Rhee EJ, Kim KH, Jo SK, Jung CH (2009). Relationship between body composition and bone mineral density (BMD) in perimenopausal Korean women. Clin Endocrinol (Oxf).

[42] Blouin K, Boivin A, Tchernof A (2008). Androgens and body fat distribution. J Steroid Biochem Mol Biol.

[43] Mayes JS, Watson GH (2004). Direct effects of sex steroid hormones on adipose tissues and obesity. Obes Rev.

[44] Pei L, Tontonoz P (2004). Fat's loss is bone's gain. J Clin Invest.

[45] Choi HS, Kim KJ, Kim KM, Hur NW, Rhee Y, Han DS (2010). Relationship between visceral adiposity and bone mineral density in Korean adults. Calcif Tissue Int.

[46] Hsu YH, Venners SA, Terwedow HA, Feng Y, Niu T, Li Z (2006). Relation of body composition, fat mass, and serum lipids to osteoporotic fractures and bone mineral density in Chinese men and women. Am J Clin Nutr.

[47] Bhupathiraju SN, Dawson-Hughes B, Hannan MT, Lichtenstein AH, Tucker KL (2011). Centrally located body fat is associated with lower bone mineral density in older Puerto Rican adults. Am J Clin Nutr.

[48] Kim HY, Choe JW, Kim HK, Bae SJ, Kim BJ, Lee SH (2010). Negative association between metabolic syndrome and bone mineral density in Koreans, especially in men. Calcif Tissue Int.

[49] Chin KY, Low NY, Dewiputri WI, Ima-Nirwanaa S (2017). Factors associated with bone health in malaysian middle-aged and elderly women assessed via quantitative ultrasound. Int J Environ Res Public Health.

[50] Sadeghi O, Saneei P, Nasiri M, Larijani B, Esmaillzadeh A (2017). Abdominal obesity and risk of hip fracture: a systematic review and meta-analysis of prospective studies. Adv Nutr.

[51] Krishnan C, Choksi P, Peterson MD (2017). Abdominal adiposity and low physical activity are independently and inversely associated with bone mineral density. Obes Res Clin Pract.

[52] Hosseini SA, Cumming RG, Bijani A, Ghadimi R, Noreddini H, Hosseini SR (2022). Relationship between visceral adipose tissue and bone mineral density in older people: Results from AHAP study. J Clin Densitom.

[53] Nanes MS (2003). Tumor necrosis factor-alpha: molecular and cellular mechanisms in skeletal pathology. Gene.

[54] Zhang P, Lin C, Chen M, He Y, Yan X, Lai J (2022). Association between visceral fat and osteoporotic vertebral compression refractures. Nutrition.

[55] Evans AL, Paggiosi MA, Eastell R, Walsh JS (2015). Bone density, microstructure and strength in obese and normal weight men and women in younger and older adulthood. J Bone Miner Res.

[56] Wang L, Wang W, Xu L, Cheng X, Ma Y, Liu D (2013). Relation of visceral and subcutaneous adipose tissue to bone mineral density in chinese women. Int J Endocrinol.

[57] Zhang X, Hua T, Zhu J, Peng K, Yang J, Kang S (2019). Body compositions differently contribute to BMD in different age and gender: a pilot study by QCT. Arch Osteoporos.

[58] Mantzoros CS, Magkos F, Brinkoetter M, Sienkiewicz E, Dardeno TA, Kim SY (2011). Leptin in human physiology and pathophysiology. Am J Physiol Endocrinol Metab.

[59] Gao L, Zhang P, Wang Y, Zhang W, Zhao J, Liu Y (2022). Relationship between body composition and bone mineral density in postmenopausal women with type 2 diabetes mellitus. BMC Musculoskelet Disord.

[60] Yamaguchi T, Kanazawa I, Yamamoto M, Kurioka S, Yamauchi M, Yano S (2009). Associations between components of the metabolic syndrome versus bone mineral density and vertebral fractures in patients with type 2 diabetes. Bone.

[61] Carvalho AL, Massaro B, Silva L, Salmon CEG, Fukada SY, Nogueira-Barbosa MH (2019). Emerging aspects of the body composition, bone marrow adipose tissue and skeletal phenotypes in type 1 diabetes mellitus. J Clin Densitom.

[62] Kawai M, de Paula FJ, Rosen CJ (2012). New insights into osteoporosis: the bone-fat connection. J Intern Med.

[63] Pahk K, Kwon Y, Kim MK, Park S, Kim S (2020). Visceral fat metabolic activity evaluated by (18)F-FDG PET/CT is associated with osteoporosis in healthy postmenopausal Korean women. Obes Res Clin Pract.

[64] Kawai T, Autieri MV, Scalia R (2021). Adipose tissue inflammation and metabolic dysfunction in obesity. Am J Physiol Cell Physiol.

[65] Charoenngam N, Apovian CM, Pongchaiyakul C (2023). Increased fat mass negatively influences femoral neck bone mineral density in men but not women. Front Endocrinol.

[66] Jain RK, Vokes T (2022). Fat mass has negative effects on bone, especially in men: a cross-sectional analysis of NHANES 2011–2018. J Clin Endocrinol Metab.

[67] Kim DH, Lim H, Chang S, Kim JN, Roh YK, Choi MK (2019). Association between Body Fat and Bone Mineral Density in Normal-Weight Middle-Aged Koreans. Korean J Fam Med.

[68] Manolopoulos KN, Karpe F, Frayn KN (2010). Gluteofemoral body fat as a determinant of metabolic health. Int J Obes (Lond).

[69] Vasan SK, Osmond C, Canoy D, Christodoulides C, Neville MJ, Di Gravio C (2018). Comparison of regional fat measurements by dual-energy X-ray absorptiometry and conventional anthropometry and their association with markers of diabetes and cardiovascular disease risk. Int J Obes (Lond).

[70] Wiklund P, Toss F, Jansson JH, Eliasson M, Hallmans G, Nordstrom A (2010). Abdominal and gynoid adipose distribution and incident myocardial infarction in women and men. Int J Obes (Lond).

[71] Toth MJ, Tchernof A, Sites CK, Poehlman ET (2000). Effect of menopausal status on body composition and abdominal fat distribution. Int J Obes Relat Metab Disord.

[72] Zamboni M, Armellini F, Milani MP, De Marchi M, Todesco T, Robbi R (1992). Body fat distribution in pre- and post-menopausal women: metabolic and anthropometric variables and their inter-relationships. Int J Obes Relat Metab Disord.

[73] Deckardt R, Lueken RP, Gallinat A, Moller CP, Busche D, Nugent W (2002). Comparison of transvaginal ultrasound, hysteroscopy, and dilatation and curettage in the diagnosis of abnormal vaginal bleeding and intrauterine pathology in perimenopausal and postmenopausal women. J Am Assoc Gynecol Laparosc.

[74] Li L, Wang Z (2018). Ovarian aging and osteoporosis. Adv Exp Med Biol.

[75] Nimrod A, Ryan KJ (1975). Aromatization of androgens by human abdominal and breast fat tissue. J Clin Endocrinol Metab.

[76] Numao S, Katayama Y, Nakata Y, Matsuo T, Nakagaichi M, Tanaka K (2020). Association of abdominal fat with metabolic syndrome components in overweight women: effect of menopausal status. J Physiol Anthropol.

