# Supplementary material for: Gender-specific associations between abdominal adipose mass and bone mineral density in the middle-aged US population
Source: BMC Musculoskelet Disord. 2023 Sep 8;24:715. doi: 10.1186/s12891-023-06844-6 (PMC10485967; doi:10.1186/s12891-023-06844-6)
Supplement: Supplementary file 1 — Additional file 1: Supplementary Table 1. Characteristics of the participants by total abdominal fat tissue mass and sex. Supplementary Table 2. Characteristics of the participants by subcutaneous adipose tissue mass and sex. Supplementary Table 3. Characteristics of the participants by visceral adipose tissue mass and sex. Supplementary Table 4. Relationships between abdominal adipose tissue and BMD stratified by menopausal status assessed by linear regression. Supplementary Table 5. Relationships between abdominal adipose tissue and the risk of low BMD stratified by menopausal status assessed by logistic regression. Supplementary Table 6. Relationships between abdominal adipose tissue and BMD stratified by obesity statuses defined by body fat percentage assessed by linear regression. [file 12891_2023_6844_MOESM1_ESM.docx]

**Supplementary Table 1** Characteristics of the participants by total abdominal fat tissue mass and sex.

|  | Men | | | | | | Women | | | | | |
| --- | --- | --- | --- | --- | --- | --- | --- | --- | --- | --- | --- | --- |
| Characteristic | Total (n=768) | Q1(n=226) | Q2(n=184) | Q3(n=206) | Q4(n=152) | P-value | Total (n=730) | Q1(n=172) | Q2(n=193) | Q3(n=178) | Q4(n=187) | P-value |
| Race |  |  |  |  |  | <.001 |  |  |  |  |  | <.001 |
| Mexican American | 28 (10.2) | 16 (5.1) | 31 (10.4) | 49 (16.7) | 28 (10.2) |  | 110 (9.2) | 5 (1.6) | 23 (8.0) | 39 (12.3) | 43 (15.1) |  |
| Other Hispanic | 13 (5.1) | 24 (7.4) | 18 (6.3) | 11 (4.0) | 13 (5.1) |  | 87 (8.0) | 21 (7.9) | 25 (9.5) | 25 (7.9) | 16 (6.8) |  |
| Non-Hispanic White | 69 (71.3) | 68 (56.9) | 59 (65.4) | 68 (58.7) | 69 (71.3) |  | 230 (59.7) | 72 (69.3) | 47 (53.9) | 51 (59.4) | 60 (56.2) |  |
| Non-Hispanic Black | 30 (9.2) | 54 (15.7) | 22 (6.3) | 41 (11.9) | 30 (9.2) |  | 146 (12.5) | 20 (7.1) | 34 (11.3) | 35 (12.5) | 57 (19.1) |  |
| Other Race | 12 (4.2) | 64 (14.9) | 54 (11.7) | 37 (8.7) | 12 (4.2) |  | 157 (10.5) | 54 (14.1) | 64 (17.3) | 28 (8.0) | 11 (2.7) |  |
| Osteoporosis |  |  |  |  |  | 0.723 |  |  |  |  |  | <.001 |
| Yes | 8 (6.9) | 20 (11.4) | 11 (6.5) | 17 (7.9) | 8 (6.9) |  | 110 (20.2) | 40 (34.9) | 30 (20.9) | 18 (11.3) | 22 (13.8) |  |
| Education |  |  |  |  |  | 0.180 |  |  |  |  |  | <.001 |
| Less than high school | 21 (9.1) | 48 (14.3) | 43 (20.8) | 49 (17.9) | 21 (9.1) |  | 140 (13.0) | 22 (9.1) | 33 (11.6) | 46 (17.8) | 39 (13.5) |  |
| High school or equivalent | 41 (30.9) | 49 (23.1) | 35 (21.5) | 49 (29.8) | 41 (30.9) |  | 151 (22.3) | 31 (14.0) | 29 (14.3) | 42 (29.5) | 49 (31.7) |  |
| College or above | 90 (60.0) | 129 (62.6) | 106 (57.7) | 108 (52.2) | 90 (60.0) |  | 439 (64.7) | 119 (77.0) | 131 (74.1) | 90 (52.7) | 99 (54.8) |  |
| Drinking |  |  |  |  |  | 0.129 |  |  |  |  |  | 0.288 |
| Nondrinker | 43 (26.5) | 52 (18.0) | 40 (13.2) | 54 (23.3) | 43 (26.5) |  | 259 (27.6) | 56 (23.3) | 62 (24.1) | 64 (32.7) | 77 (30.5) |  |
| Low-to-moderate drinker | 88 (60.6) | 120 (58.0) | 116 (68.3) | 108 (55.6) | 88 (60.6) |  | 355 (57.6) | 81 (56.5) | 96 (59.2) | 88 (55.9) | 90 (58.7) |  |
| Heavy drinker | 17 (9.8) | 24 (14.7) | 16 (10.8) | 21 (14.4) | 17 (9.8) |  | 47 (8.0) | 11 (8.6) | 15 (10.8) | 13 (8.0) | 8 (4.6) |  |
| Smoking |  |  |  |  |  | 0.038 |  |  |  |  |  | 0.670 |
| Never smoker | 77 (48.9) | 118 (57.7) | 106 (62.4) | 107 (55.0) | 77 (48.9) |  | 509 (65.4) | 122 (72.1) | 144 (66.4) | 121 (59.0) | 122 (64.0) |  |
| Current smoker | 31 (19.2) | 66 (28.5) | 42 (20.5) | 43 (18.5) | 31 (19.2) |  | 111 (14.9) | 28 (14.3) | 22 (13.0) | 31 (19.0) | 30 (13.4) |  |
| Ever smoker | 44 (32.0) | 42 (13.8) | 36 (17.1) | 56 (26.6) | 44 (32.0) |  | 110 (19.7) | 22 (13.6) | 27 (20.6) | 26 (22.0) | 35 (22.6) |  |
| Leisure activity |  |  |  |  |  | 0.004 |  |  |  |  |  | <.001 |
| No leisure-time physical activity | 98 (58.7) | 107 (41.8) | 106 (56.7) | 126 (61.9) | 98 (58.7) |  | 383 (46.8) | 73 (30.9) | 96 (40.9) | 99 (54.0) | 115 (61.5) |  |
| Insufficiently active-moderate activity | 43 (33.4) | 70 (33.0) | 47 (25.4) | 54 (25.9) | 43 (33.4) |  | 230 (35.9) | 56 (39.5) | 66 (37.8) | 55 (36.1) | 53 (30.3) |  |
| Active-moderate activity | 11 (7.9) | 49 (25.3) | 31 (17.9) | 26 (12.2) | 11 (7.9) |  | 116 (17.2) | 43 (29.6) | 30 (21.0) | 24 (9.9) | 19 (8.2) |  |
| Calcium supplement use |  |  |  |  |  | 0.905 |  |  |  |  |  | 0.001 |
| Yes | 49 (35.8) | 77 (38.4) | 67 (41.1) | 68 (38.8) | 49 (35.8) |  | 297 (46.9) | 83 (54.8) | 91 (58.4) | 60 (40.3) | 63 (34.1) |  |
| Vitamin D supplement use |  |  |  |  |  | 0.451 |  |  |  |  |  | 0.009 |
| Yes | 39 (28.8) | 71 (37.5) | 67 (41.1) | 63 (35.3) | 39 (28.8) |  | 308 (48.9) | 84 (55.3) | 95 (59.0) | 65 (40.2) | 64 (41.0) |  |
| Hormone replacement therapy |  |  |  |  |  | - |  |  |  |  |  | 0.920 |
| Yes | - | - | - | - | - |  | 83 (14.8) | 19 (12.7) | 20 (15.0) | 22 (15.9) | 22 (15.3) |  |
| Diabetes |  |  |  |  |  | <.001 |  |  |  |  |  | <.001 |
| Yes | 44 (24.6) | 14 (5.7) | 31 (11.3) | 41 (13) | 44 (24.6) |  | 102 (9.1) | 5 (1.5) | 20 (6.0) | 24 (6.0) | 53 (22.8) |  |
| Family income-to-poverty ratio |  |  |  |  |  | 0.067 |  |  |  |  |  | <.001 |
| 0-1.0 | 21 (9.4) | 55 (18.4) | 20 (8.3) | 33 (9.1) | 21 (9.4) |  | 119 (10.7) | 26 (7.9) | 20 (6.5) | 35 (14.7) | 38 (13.8) |  |
| 1.0-3.0 | 47 (23.7) | 62 (25.3) | 69 (37.0) | 59 (31) | 47 (23.7) |  | 234 (26.0) | 36 (15.7) | 56 (21.5) | 67 (31.6) | 75 (35.3) |  |
| >3.0 | 70 (61.0) | 90 (49.7) | 78 (46.2) | 91 (52.2) | 70 (61.0) |  | 317 (55.4) | 96 (67.7) | 102 (65.0) | 58 (42.9) | 61 (45.7) |  |
| Menopause |  |  |  |  |  |  |  |  |  |  |  | 0.279 |
| Yes |  |  |  |  |  |  | 328 (48.3) | 64 (38.5) | 90 (50.6) | 85 (52.3) | 89 (51.8) |  |
| Age, years | 49.3(48.7 - 50.0) | 48.8 ((47.8 - 49.9)) | 49.9(49.0 - 50.8) | 49.0(48.1 - 49.9) | 49.6(48.8 - 50.5) | 0.264 | 730 (49.7) | 48.5(47.0 - 50.0) | 50.4(49.1 - 51.7) | 50.3(49.0 - 51.6) | 187 (49.7) | 0.154 |
| BMI, kg/m^2^ | 29.0(28.5 - 29.6) | 23.9(23.4 - 24.4) | 27.4(26.7 - 28.1) | 29.4(28.9 - 29.9) | 35.4(34.5 - 36.4) | <.001 | 28.3(27.7 - 28.8) | 21.7(21.3 - 22.1) | 25.2(24.6 - 25.8) | 29.4(28.9 - 30.0) | 36.7(36.1 - 37.3) | <.0001 |
| Total calcium, mmol/L | 2.35(2.34 - 2.36) | 2.36(2.34 - 2.38) | 2.34(2.32 - 2.36) | 2.34(2.33 - 2.36) | 2.34(2.33 - 2.35) | 0.176 | 2.35(2.34 - 2.36) | 2.35(2.33 - 2.36) | 2.36(2.34 - 2.39) | 2.36(2.34 - 2.38) | 2.34(2.32 - 2.36) | 0.522 |
| Phosphorus, mmol/L | 1.17(1.15 - 1.19) | 1.19(1.15 - 1.22) | 1.15(1.11 - 1.18) | 1.14(1.11 - 1.18) | 1.2(1.16 - 1.23) | <.001 | 1.23(1.21 - 1.25) | 1.27(1.24 - 1.31) | 1.23(1.19 - 1.28) | 1.21(1.17 - 1.25) | 1.20(1.18 - 1.23) | 0.004 |
| Subcutaneous adipose tissue mass, kg | 1.37(1.31 - 1.44) | 0.71(0.66 - 0.76) | 1.15(1.11 - 1.20) | 1.49(1.46 - 1.51) | 2.15(2.05 - 2.25) | <.001 | 1.82(1.76 - 1.89) | 1.02(0.97 - 1.07) | 1.56(1.53 - 1.60) | 1.98(1.94 - 2.01) | 2.74(2.66 - 2.81) | <.0001 |
| Total abdominal fat tissue mass, kg | 2.02(1.94 - 2.10) | 1.07(1.01 - 1.13) | 1.70(1.67 - 1.73) | 2.19(2.16 - 2.22) | 3.14(3.01 - 3.27) | <.001 | 2.33(2.24 - 2.41) | 1.24(1.17 - 1.30) | 1.96(1.92 - 2.00) | 2.57(2.53 - 2.60) | 3.55(3.49 - 3.61) | <.0001 |
| Visceral adipose tissue mass, kg | 0.65(0.62 - 0.67) | 0.36(0.34 - 0.38) | 0.54(0.52 - 0.57) | 0.70(0.68 - 0.72) | 0.99(0.93 - 1.05) | <.001 | 0.50(0.48 - 0.53) | 0.21(0.20 - 0.23) | 0.40(0.37 - 0.42) | 0.59(0.56 - 0.62) | 0.82(0.77 - 0.86) | <.0001 |
| Femoral neck BMD, gm/cm2 | 0.83(0.82 - 0.85) | 0.79(0.77 - 0.82) | 0.83(0.80 - 0.86) | 0.84(0.81 - 0.86) | 0.87(0.84 - 0.89) | 0.005 | 0.79(0.78 - 0.80) | 0.73(0.71 - 0.75) | 0.76(0.73 - 0.79) | 0.79(0.76 - 0.81) | 0.87(0.85 - 0.88) | <.0001 |
| Lumbar spine BMD, gm/cm2 | 1.04(1.03 - 1.05) | 1.00(0.97 - 1.03) | 1.03(1.00 - 1.06) | 1.06(1.04 - 1.08) | 1.07(1.04 - 1.11) | 0.002 | 1.02(1.00 - 1.03) | 0.99(0.96 - 1.01) | 0.99(0.96 - 1.02) | 1.00(0.97 - 1.03) | 1.08(1.06 - 1.10) | 0.001 |

Data were presented as n (weighted percentage) for categorical variables and weighted mean (95% CI) for continuous variables. **Abbreviations:** BMD bone mineral density, BMI body mass index, CI: confidence interval.

**Supplementary Table 2** Characteristics of the participants by subcutaneous adipose tissue mass and sex.

|  | Men | | | | | | Women | | | | | |
| --- | --- | --- | --- | --- | --- | --- | --- | --- | --- | --- | --- | --- |
| Characteristic | Total(n=768) | Q1(n=220) | Q2(n=210) | Q3(n=169) | Q4(n=169) | P-value | Total(n=730) | Q1(n=183) | Q2(n=187) | Q3(n=174) | Q4(n=186) | P-value |
| Race |  |  |  |  |  | <.001 |  |  |  |  |  | <.001 |
| Mexican American | 124 (10.6) | 18 (5.6) | 43 (14.8) | 33 (10.7) | 30 (11.4) |  | 110 (9.2) | 7 (2.3) | 27 (9.3) | 34 (10.7) | 42 (14.7) |  |
| Other Hispanic | 66 (5.7) | 21 (6.2) | 24 (8.3) | 5 (1.7) | 16 (6.4) |  | 87 (8.0) | 24 (9.2) | 26 (9.2) | 23 (8.8) | 14 (5.0) |  |
| Non-Hispanic White | 264 (63.0) | 70 (61.0) | 58 (56.5) | 69 (70.8) | 67 (63.9) |  | 230 (59.7) | 69 (65.7) | 49 (55.1) | 53 (60.9) | 59 (57.0) |  |
| Non-Hispanic Black | 147 (10.8) | 46 (13.4) | 22 (5.9) | 36 (10.8) | 43 (13.1) |  | 146 (12.5) | 21 (7.4) | 29 (9.6) | 36 (12.7) | 60 (20.3) |  |
| Other Race | 167 (9.9) | 65 (13.7) | 63 (14.5) | 26 (5.9) | 13 (5.3) |  | 157 (10.5) | 62 (15.4) | 56 (16.8) | 28 (6.9) | 11 (3.0) |  |
| Osteoporosis |  |  |  |  |  | 0.549 |  |  |  |  |  | <.001 |
| Yes | 56 (8.2) | 21 (11.3) | 13 (7.4) | 12 (7.0) | 10 (6.9) |  | 110 (20.2) | 41 (35.1) | 29 (20.8) | 19 (10.0) | 21 (14.9) |  |
| Education |  |  |  |  |  | 0.340 |  |  |  |  |  | 0.001 |
| Less than high school | 161 (15.6) | 53 (18.4) | 50 (19.2) | 29 (12.1) | 29 (12.4) |  | 140 (13.0) | 24 (9.7) | 39 (13.1) | 41 (16.9) | 36 (12.4) |  |
| High school or equivalent | 174 (26.3) | 48 (27.1) | 41 (19.1) | 43 (30.7) | 42 (28.5) |  | 151 (22.3) | 31 (13.8) | 31 (15.1) | 41 (30.0) | 48 (30.4) |  |
| College or above | 433 (58.1) | 119 (54.5) | 119 (61.7) | 97 (57.3) | 98 (59.1) |  | 439 (64.7) | 128 (76.5) | 117 (71.8) | 92 (53.1) | 102 (57.3) |  |
| Drinking |  |  |  |  |  | 0.024 |  |  |  |  |  | 0.074 |
| Nondrinker | 189 (20.3) | 49 (16.2) | 50 (17.3) | 40 (17.0) | 50 (30.5) |  | 259 (27.6) | 63 (24.8) | 59 (22.6) | 65 (34.4) | 72 (28.8) |  |
| Low-to-moderate drinker | 432 (60.6) | 116 (59.6) | 120 (58.6) | 99 (66.1) | 97 (58.3) |  | 355 (57.6) | 86 (55.3) | 89 (58.8) | 87 (57.0) | 93 (59.2) |  |
| Heavy drinker | 78 (12.4) | 25 (14.6) | 20 (14.4) | 17 (12.1) | 16 (8.6) |  | 47 (8.0) | 11 (8.6) | 17 (12.3) | 12 (5.9) | 7 (5.1) |  |
| Smoking |  |  |  |  |  | 0.139 |  |  |  |  |  | 0.735 |
| Never smoker | 408 (56.0) | 116 (58.4) | 116 (57.8) | 89 (54.3) | 87 (53.3) |  | 509 (65.4) | 134 (72.8) | 138 (66.0) | 117 (59.2) | 120 (63.5) |  |
| Current smoker | 182 (21.7) | 64 (26.6) | 48 (23.9) | 36 (17.0) | 34 (19.1) |  | 111 (14.9) | 27 (13.9) | 22 (13.3) | 30 (17.6) | 32 (15.0) |  |
| Ever smoker | 178 (22.3) | 40 (15.0) | 46 (18.3) | 44 (28.7) | 48 (27.6) |  | 110 (19.7) | 22 (13.3) | 27 (20.7) | 27 (23.3) | 34 (21.4) |  |
| Leisure activity |  |  |  |  |  | 0.184 |  |  |  |  |  | <.001 |
| No leisure-time physical activity | 437 (54.8) | 112 (48.9) | 123 (54.5) | 95 (54.8) | 107 (60.8) |  | 383 (46.8) | 83 (32.9) | 93 (41.4) | 95 (52.3) | 112 (60.8) |  |
| Insufficiently active-moderate activity | 214 (29.4) | 61 (27.5) | 57 (28.0) | 47 (31.5) | 49 (30.8) |  | 230 (35.9) | 57 (38.4) | 63 (36.8) | 53 (36.1) | 57 (32.4) |  |
| Active-moderate activity | 117 (15.8) | 47 (23.6) | 30 (17.5) | 27 (13.7) | 13 (8.4) |  | 116 (17.2) | 43 (28.7) | 31 (21.8) | 25 (11.4) | 17 (6.8) |  |
| Calcium supplement use |  |  |  |  |  | 0.425 |  |  |  |  |  | 0.014 |
| Yes | 261 (38.5) | 73 (35.3) | 76 (45.0) | 61 (38.5) | 51 (35.5) |  | 297 (46.9) | 86 (55.7) | 81 (52.3) | 71 (45.4) | 59 (34.2) |  |
| Vitamin D supplement use |  |  |  |  |  | 0.337 |  |  |  |  |  | 0.106 |
| Yes | 240 (35.7) | 70 (36.0) | 71 (42.5) | 54 (36.7) | 45 (27.5) |  | 308 (48.9) | 92 (57.2) | 82 (52.7) | 68 (43.1) | 66 (42.7) |  |
| Hormone replacement therapy |  |  |  |  |  |  |  |  |  |  |  | 0.482 |
| Yes |  |  |  |  |  |  | 83 (14.8) | 20 (12.8) | 17 (12.4) | 24 (19.4) | 22 (14.4) |  |
| Diabetes |  |  |  |  |  | <.001 |  |  |  |  |  | <.001 |
| Yes | 130 (13.6) | 19 (8.9) | 32 (9.8) | 32 (11.8) | 47 (24.2) |  | 102 (9.1) | 7 (2.0) | 19 (5.5) | 24 (6.4) | 52 (22.4) |  |
| Family income-to-poverty ratio |  |  |  |  |  | 0.055 |  |  |  |  |  | 0.008 |
| 0-1.0 | 129 (11.3) | 54 (18.4) | 30 (10.6) | 22 (6.6) | 23 (9.4) |  | 119 (10.7) | 27 (8.1) | 23 (7.5) | 31 (13.9) | 38 (13.3) |  |
| 1.0-3.0 | 237 (29.2) | 64 (30.2) | 65 (28.8) | 56 (31.5) | 52 (26.4) |  | 234 (26.0) | 41 (16.7) | 54 (21.6) | 66 (31.2) | 73 (34.7) |  |
| >3.0 | 329 (52.3) | 82 (44.4) | 93 (50.6) | 74 (54.8) | 80 (59.4) |  | 317 (55.4) | 100 (66.1) | 93 (63.2) | 60 (44.6) | 64 (47.5) |  |
| Menopause |  |  |  |  |  |  |  |  |  |  |  | 0.238 |
| Yes |  |  |  |  |  |  | 328 (48.3) | 71 (40.0) | 83 (47.5) | 90 (56.2) | 84 (49.5) |  |
| Age, years | 49.3(48.7 - 50.0) | 49.7(48.4 - 50.9) | 49.4(48.6 - 50.3) | 49.5(48.2 - 50.8) | 48.8(48.0 - 49.6) | 0.459 | 49.7(49.0 - 50.5) | 48.7(47.3 - 50.1) | 50.4(49.0 - 51.8) | 50.2(48.8 - 51.6) | 49.6(48.7 - 50.6) | 0.180 |
| BMI, kg/m^2^ | 29.0(28.5 - 29.6) | 23.9(23.4 - 24.5) | 27.1(26.6 - 27.6) | 29.7(28.9 - 30.4) | 35.5(34.6 - 36.3) | <.001 | 28.3(27.7 - 28.8) | 21.8(21.4 - 22.2) | 25.3(24.6 - 26.0) | 29.2(28.7 - 29.8) | 36.7(36.1 - 37.3) | <.001 |
| Total calcium, mmol/L | 2.35(2.34 - 2.36) | 2.35(2.34 - 2.37) | 2.35(2.33 - 2.37) | 2.35(2.33 - 2.36) | 2.34(2.32 - 2.35) | 0.269 | 2.35(2.34 - 2.36) | 2.35(2.33 - 2.36) | 2.36(2.34 - 2.39) | 2.36(2.34 - 2.37) | 2.34(2.33 - 2.36) | 0.335 |
| Phosphorus, mmol/L | 1.17(1.15 - 1.19) | 1.16(1.13 - 1.20) | 1.16(1.14 - 1.18) | 1.17(1.14 - 1.20) | 1.18(1.15 - 1.21) | 0.762 | 1.23(1.21 - 1.25) | 1.27(1.23 - 1.30) | 1.24(1.20 - 1.28) | 1.21(1.18 - 1.24) | 1.2(1.18 - 1.22) | 0.002 |
| Subcutaneous adipose tissue mass, kg | 1.37(1.31 - 1.44) | 0.70(0.66 - 0.75) | 1.14(1.13 - 1.16) | 1.49(1.47 - 1.51) | 2.17(2.10 - 2.24) | <.001 | 1.82(1.76 - 1.89) | 1.02(0.98 - 1.06) | 1.55(1.52 - 1.57) | 1.98(1.95 - 2.01) | 2.76(2.68 - 2.83) | <.001 |
| Total abdominal fat tissue mass, kg | 2.02(1.94 - 2.10) | 1.10(1.03 - 1.17) | 1.70(1.67 - 1.73) | 2.21(2.15 - 2.27) | 3.10(2.98 - 3.22) | <.001 | 2.33(2.24 - 2.41) | 1.24(1.19 - 1.30) | 1.98(1.92 - 2.04) | 2.56(2.50 - 2.61) | 3.54(3.47 - 3.61) | <.001 |
| Visceral adipose tissue mass, kg | 0.65(0.62 - 0.67) | 0.40(0.37 - 0.43) | 0.55(0.53 - 0.57) | 0.72(0.67 - 0.77) | 0.92(0.86 - 0.98) | <.001 | 0.50(0.48 - 0.53) | 0.22(0.21 - 0.24) | 0.43(0.40 - 0.47) | 0.58(0.54 - 0.61) | 0.78(0.73 - 0.83) | <.001 |
| Femoral neck BMD, gm/cm2 | 0.83(0.82 - 0.85) | 0.79(0.77 - 0.82) | 0.82(0.79 - 0.86) | 0.83(0.80 - 0.85) | 0.88(0.86 - 0.90) | <.001 | 0.79(0.78 - 0.80) | 0.73(0.71 - 0.75) | 0.75(0.72 - 0.77) | 0.80(0.77 - 0.82) | 0.87(0.86 - 0.88) | <.001 |
| Lumbar spine BMD, gm/cm2 | 1.04(1.03 - 1.05) | 1.00(0.97 - 1.03) | 1.03(1.00 - 1.06) | 1.04(1.00 - 1.08) | 1.09(1.06 - 1.12) | 0.002 | 1.02(1.00 - 1.03) | 0.98(0.96 - 1.01) | 0.99(0.96 - 1.01) | 1.01(0.97 - 1.04) | 1.09(1.07 - 1.11) | <.001 |

Data were presented as n (weighted percentage) for categorical variables and weighted mean (95% CI) for continuous variables. **Abbreviations:** BMD bone mineral density, BMI body mass index, CI: confidence interval.

**Supplementary Table 3** Characteristics of the participants by visceral adipose tissue mass and sex.

|  |  | Men | | | | | Women | | | | | |
| --- | --- | --- | --- | --- | --- | --- | --- | --- | --- | --- | --- | --- |
| Characteristic | Total (n=768) | Q1(n=222) | Q2(n=202) | Q3(n=181) | Q4(n=163) | P-value | Total(n=730) | Q1(n=159) | Q2(n=192) | Q3(n=192) | Q4(n=187) | P-value |
| Race |  |  |  |  |  | <.001 |  |  |  |  |  | <.001 |
| Mexican American | 124 (10.6) | 12 (4.0) | 36 (12.9) | 39 (13.2) | 37 (12.2) |  | 110 (9.2) | 4 (1.5) | 18 (6.4) | 36 (11.5) | 52 (17.5) |  |
| Other Hispanic | 66 (5.7) | 18 (5.3) | 19 (6.6) | 11 (4.1) | 18 (6.7) |  | 87 (8.0) | 17 (6.7) | 24 (8.5) | 25 (8.4) | 21 (8.6) |  |
| Non-Hispanic White | 264 (63.0) | 64 (56.9) | 59 (57.3) | 66 (66.3) | 75 (71.7) |  | 230 (59.7) | 69 (69.6) | 50 (55.7) | 53 (56.1) | 58 (57.4) |  |
| Non-Hispanic Black | 147 (10.8) | 68 (19.2) | 38 (11.6) | 22 (7.2) | 19 (5.2) |  | 146 (12.5) | 31 (10.8) | 44 (14.9) | 41 (13.8) | 30 (10.5) |  |
| Other Race | 167 (9.9) | 60 (14.5) | 50 (11.5) | 43 (9.2) | 14 (4.2) |  | 157 (10.5) | 38 (11.3) | 56 (14.5) | 37 (10.3) | 26 (6.0) |  |
| Osteoporosis |  |  |  |  |  | 0.127 |  |  |  |  |  | 0.006 |
| Yes | 56 (8.2) | 17 (8.3) | 17 (12.4) | 12 (4.4) | 10 (7.7) |  | 110 (20.2) | 33 (29.4) | 29 (24.8) | 28 (14.9) | 20 (11.8) |  |
| Education |  |  |  |  |  | 0.584 |  |  |  |  |  | <.001 |
| Less than high school | 161 (15.6) | 45 (14.4) | 42 (15.3) | 39 (19.3) | 35 (13.2) |  | 140 (13.0) | 19 (7.9) | 26 (10.0) | 43 (14.4) | 52 (19.7) |  |
| High school or equivalent | 174 (26.3) | 49 (22.2) | 40 (23.5) | 46 (28.6) | 39 (31.1) |  | 151 (22.3) | 27 (13.9) | 30 (18.1) | 46 (25.9) | 48 (31.5) |  |
| College or above | 433 (58.1) | 128 (63.4) | 120 (61.2) | 96 (52.1) | 89 (55.7) |  | 439 (64.7) | 113 (78.2) | 136 (71.9) | 103 (59.7) | 87 (48.8) |  |
| Drinking |  |  |  |  |  | 0.207 |  |  |  |  |  | 0.296 |
| Nondrinker | 189 (20.3) | 55 (19.0) | 41 (13.5) | 49 (21.6) | 44 (26.9) |  | 259 (27.6) | 49 (20.6) | 61 (26.7) | 76 (32.4) | 73 (30.8) |  |
| Low-to-moderate drinker | 432 (60.6) | 115 (57.9) | 131 (69.6) | 98 (56.5) | 88 (58.4) |  | 355 (57.6) | 80 (59.3) | 98 (57.1) | 85 (54.8) | 92 (59.0) |  |
| Heavy drinker | 78 (12.4) | 22 (13.6) | 15 (9.6) | 21 (16.9) | 20 (9.6) |  | 47 (8.0) | 11 (10.3) | 14 (10.0) | 12 (5.8) | 10 (5.9) |  |
| Smoking |  |  |  |  |  | <.001 |  |  |  |  |  | 0.262 |
| Never smoker | 408 (56.0) | 117 (57.6) | 123 (65.8) | 97 (52.7) | 71 (47.9) |  | 509 (65.4) | 108 (70.4) | 144 (65.3) | 130 (60.7) | 127 (65.1) |  |
| Current smoker | 182 (21.7) | 67 (30.0) | 39 (17.2) | 41 (22.5) | 35 (16.9) |  | 111 (14.9) | 26 (14.2) | 27 (19.0) | 29 (11.1) | 29 (15.4) |  |
| Ever smoker | 178 (22.3) | 38 (12.4) | 40 (17) | 43 (24.8) | 57 (35.3) |  | 110 (19.7) | 25 (15.4) | 21 (15.7) | 33 (28.2) | 31 (19.5) |  |
| Leisure activity |  |  |  |  |  | 0.005 |  |  |  |  |  | <.001 |
| No leisure-time physical activity | 437 (54.8) | 105 (39.7) | 124 (59.3) | 103 (62.9) | 105 (57.0) |  | 383 (46.8) | 64 (29.8) | 90 (37.5) | 104 (53) | 125 (67.0) |  |
| Insufficiently active-moderate activity | 214 (29.4) | 67 (34.1) | 51 (24.5) | 51 (26.0) | 45 (33.1) |  | 230 (35.9) | 49 (36.2) | 73 (47.0) | 64 (35.3) | 44 (25.2) |  |
| Active-moderate activity | 117 (15.8) | 50 (26.1) | 27 (16.2) | 27 (11.1) | 13 (9.9) |  | 116 (17.2) | 45 (33.8) | 29 (15.5) | 24 (11.7) | 18 (7.8) |  |
| Calcium supplement use |  |  |  |  |  | 0.944 |  |  |  |  |  | 0.002 |
| Yes | 261 (38.5) | 78 (40.9) | 67 (38.7) | 61 (37.9) | 55 (36.6) |  | 297 (46.9) | 69 (49.9) | 94 (58.8) | 74 (45.8) | 60 (33.2) |  |
| Vitamin D supplement use |  |  |  |  |  | 0.397 |  |  |  |  |  | 0.039 |
| Yes | 240 (35.7) | 72 (39.1) | 68 (40.6) | 52 (30.3) | 48 (32.7) |  | 308 (48.9) | 73 (52.3) | 98 (58.7) | 74 (42.4) | 63 (42.2) |  |
| Hormone replacement therapy |  |  |  |  |  |  |  |  |  |  |  | 0.189 |
| Yes |  |  |  |  |  |  | 83 (14.8) | 15 (10.6) | 28 (19.7) | 15 (11.1) | 25 (17.6) |  |
| Diabetes |  |  |  |  |  | <.001 |  |  |  |  |  | <.001 |
| Yes | 130 (13.6) | 19 (6.8) | 24 (8) | 40 (14.9) | 47 (25.0) |  | 102 (9.1) | 6 (2.0) | 12 (4.5) | 37 (10.4) | 47 (19.5) |  |
| Family income-to-poverty ratio |  |  |  |  |  | 0.286 |  |  |  |  |  | <.001 |
| 0-1.0 | 129 (11.3) | 49 (15.7) | 26 (7.9) | 25 (10.8) | 29 (10.8) |  | 119 (10.7) | 20 (6.2) | 25 (11.9) | 33 (10.1) | 41 (14.5) |  |
| 1.0-3.0 | 237 (29.2) | 60 (25.8) | 67 (32.5) | 58 (34.6) | 52 (23.9) |  | 234 (26.0) | 38 (17.2) | 52 (19.9) | 64 (27.6) | 80 (39.5) |  |
| >3.0 | 329 (52.3) | 91 (49.7) | 91 (52.1) | 80 (47.9) | 67 (59.5) |  | 317 (55.4) | 91 (69.1) | 101 (62.1) | 77 (51.6) | 48 (38.5) |  |
| Menopause |  |  |  |  |  |  |  |  |  |  |  | <.001 |
| Yes |  |  |  |  |  |  | 328 (48.3) | 47 (29.0) | 89 (55.6) | 90 (48.8) | 102 (59.8) |  |
| Age, years | 49.3(48.7 - 50.0) | 48.3(47.2 - 49.4) | 49.6(48.5 - 50.7) | 49.2(48.2 - 50.3) | 50.3(49.3 - 51.3) | 0.048 | 49.7(49.0 - 50.5) | 47.8(46.6 - 49.0) | 50.3(49.0 - 51.6) | 50(49.0 - 51.1) | 50.8(49.8 - 51.8) | 0.003 |
| BMI, kg/m^2^ | 29(28.5 - 29.6) | 24.8(24.1 - 25.5) | 27.6(27.1 - 28.2) | 29.5(28.8 - 30.3) | 34.2(33.0 - 35.3) | <.001 | 28.3(27.7 - 28.8) | 22.3(21.8 - 22.8) | 25.9(25.1 - 26.7) | 30.3(29.4 - 31.2) | 34.5(33.7 - 35.2) | <.001 |
| Total calcium, mmol/L | 2.35(2.34 - 2.36) | 2.36(2.34 - 2.37) | 2.34(2.32 - 2.36) | 2.35(2.33 - 2.36) | 2.34(2.32 - 2.35) | 0.228 | 2.35(2.34 - 2.36) | 2.34(2.32 - 2.35) | 2.37(2.35 - 2.39) | 2.35(2.33 - 2.37) | 2.35(2.33 - 2.37) | 0.037 |
| Phosphorus, mmol/L | 1.17(1.15 - 1.19) | 1.19(1.15 - 1.23) | 1.14(1.11 - 1.18) | 1.18(1.15 - 1.20) | 1.16(1.12 - 1.20) | 0.395 | 1.23(1.21 - 1.25) | 1.28(1.24 - 1.31) | 1.21(1.17 - 1.26) | 1.22(1.18 - 1.25) | 1.21(1.18 - 1.23) | 0.006 |
| Subcutaneous adipose tissue mass, kg | 1.37(1.31 - 1.44) | 0.83(0.75 - 0.91) | 1.25(1.16 - 1.33) | 1.49(1.41 - 1.57) | 1.94(1.82 - 2.07) | <.001 | 1.82(1.76 - 1.89) | 1.13(1.04 - 1.22) | 1.67(1.57 - 1.76) | 2.08(1.98 - 2.18) | 2.42(2.34 - 2.49) | <.001 |
| Total abdominal fat tissue mass, kg | 2.02(1.94 - 2.10) | 1.16(1.08 - 1.25) | 1.77(1.68 - 1.85) | 2.18(2.10 - 2.26) | 2.98(2.82 - 3.15) | <.001 | 2.33(2.24 - 2.41) | 1.33(1.23 - 1.42) | 2.04(1.94 - 2.14) | 2.64(2.54 - 2.75) | 3.3(3.22 - 3.38) | <.001 |
| Visceral adipose tissue mass, kg | 0.65(0.62 - 0.67) | 0.33(0.32 - 0.35) | 0.52(0.51 - 0.53) | 0.69(0.68 - 0.70) | 1.04(0.99 - 1.10) | <.001 | 0.50(0.48 - 0.53) | 0.19(0.18 - 0.20) | 0.37(0.36 - 0.39) | 0.56(0.55 - 0.58) | 0.88(0.85 - 0.92) | <.001 |
| Femoral neck BMD, gm/cm2 | 0.83(0.82 - 0.85) | 0.81(0.79 - 0.83) | 0.83(0.80 - 0.87) | 0.83(0.82 - 0.85) | 0.85(0.82 - 0.87) | 0.288 | 0.79(0.78 - 0.80) | 0.75(0.72 - 0.77) | 0.78(0.75 - 0.80) | 0.79(0.77 - 0.81) | 0.83(0.81 - 0.85) | <.001 |
| Lumbar spine BMD, gm/cm2 | 1.04(1.03 - 1.05) | 1.01(0.98 - 1.04) | 1.04(1.01 - 1.07) | 1.04(1.03 - 1.06) | 1.06(1.03 - 1.10) | 0.164 | 1.02(1.00 - 1.03) | 1.00(0.97 - 1.03) | 1.00(0.98 - 1.03) | 1.02(0.99 - 1.05) | 1.04(1.01 - 1.06) | 0.288 |

Description: Data were presented as n (weighted percentage) for categorical variables and weighted mean (95% CI) for continuous variables. **Abbreviations:** BMD bone mineral density, BMI body mass index, CI: confidence interval.

**Supplementary Table 4** Relationships between abdominal adipose tissue and BMD stratified by menopausal status assessed by linear regression.

|  | Femoral neck BMD | | | | Lumbar spine BMD | | | |
| --- | --- | --- | --- | --- | --- | --- | --- | --- |
|  | Univariate model | | Multivariate model | | Univariate model | | Multivariate model | |
|  | Coefficient (95% CI) | P value | Coefficient (95% CI) | P value | Coefficient (95% CI) | P value | Coefficient (95% CI) | P value |
| TAT |  |  |  |  |  |  |  |  |
| Non-menopause | 0.073 (0.060-0.086) | <0.001 | -0.022 (-0.06-0.015) | 0.235 | 0.044 (0.026-0.061) | <0.001 | 0.010 (-0.035-0.055) | 0.653 |
| Menopause | 0.056 (0.037-0.075) | <0.001 | -0.002 (-0.051-0.048) | 0.948 | 0.051 (0.023-0.08) | 0.001 | -0.008 (-0.071-0.056) | 0.806 |
| SAT |  |  |  |  |  |  |  |  |
| Non-menopause | 0.092 (0.077-0.107) | <0.001 | -0.036 (-0.088-0.016) | 0.163 | 0.057 (0.036-0.078) | <0.001 | 0.018 (-0.029-0.064) | 0.441 |
| Menopause | 0.079 (0.054-0.103) | <0.001 | 0.003 (-0.052-0.059) | 0.899 | 0.073 (0.038-0.108) | <0.001 | 0.000 (-0.065-0.064) | 0.994 |
| VAT |  |  |  |  |  |  |  |  |
| Non-menopause | 0.190 (0.129-0.252) | <0.001 | 0.014 (-0.058-0.087) | 0.687 | 0.101 (0.04-0.161) | 0.002 | -0.010 (-0.088-0.068) | 0.794 |
| Menopause | 0.133 (0.058-0.208) | 0.001 | -0.015 (-0.144-0.114) | 0.809 | 0.120 (0.014-0.225) | 0.028 | -0.031 (-0.169-0.106) | 0.645 |

Linear regression models were used to estimate the regression coefficients and 95% CI for the association of menopausal status with BMD. **Abbreviations:** BMD bone mineral density, CI: confidence interval，SAT subcutaneous adipose tissue, TAT total adipose tissue, VAT visceral adipose tissue.

**Supplementary Table 5** Relationships between abdominal adipose tissue and the risk of low BMD stratified by menopausal status assessed by logistic regression.

|  | Femoral neck low BMD | | | | Lumbar spine low BMD | | | |
| --- | --- | --- | --- | --- | --- | --- | --- | --- |
|  | Univariate model | | Multivariate model | | Univariate model | | Multivariate model | |
|  | OR  (95%CI) | P value | OR  (95%CI) | P value | OR  (95%CI) | P value | OR  (95%CI) | P value |
| TAT |  |  |  |  |  |  |  |  |
| Non-menopause | 0.30  (0.20-0.45) | <0.001 | 2.01  (0.55-7.29) | 0.27 | 0.61  (0.36-1.04) | 0.06 | 1.70  (0.53-5.45) | 0.352 |
| Menopause | 0.42  (0.27-0.63) | <0.001 | 0.89  (0.31-2.49) | 0.809 | 0.62  (0.43-0.88) | 0.006 | 0.75  (0.3-1.87) | 0.524 |
| SAT |  |  |  |  |  |  |  |  |
| Non-menopause | 0.22  (0.13-0.37) | <0.001 | 3.94  (0.72-21.47) | 0.098 | 0.50  (0.24-1.04) | 0.052 | 1.48  (0.42-5.17) | 0.52 |
| Menopause | 0.28  (0.16-0.49) | <0.001 | 0.69  (0.17-2.85) | 0.594 | 0.46  (0.27-0.77) | 0.002 | 0.38  (0.1-1.43) | 0.134 |
| VAT |  |  |  |  |  |  |  |  |
| Non-menopause | 0.02  (0.00-0.11) | <0.001 | 0.35  (0.02-6.45) | 0.462 | 0.35  (0.07-1.82) | 0.195 | 2.76  (0.14-53.05) | 0.483 |
| Menopause | 0.14  (0.03-0.65) | 0.009 | 1.47  (0.09-25.27) | 0.781 | 0.50  (0.18-1.41) | 0.17 | 3.71  (0.68-20.23) | 0.114 |

Logistic regression models were used to estimate OR and 95% CI for the association of menopausal status with the risk of low bone mineral density (BMD). **Abbreviations:** BMD bone mineral density, CI: confidence interval，OR odd ratio, SAT subcutaneous adipose tissue, TAT total adipose tissue, VAT visceral adipose tissue.

**Supplementary Table 6** Relationships between abdominal adipose tissue and BMD stratified by obesity statuses defined by body fat percentage assessed by linear regression

|  | Femoral neck BMD | | | | Lumbar spine BMD | | | |
| --- | --- | --- | --- | --- | --- | --- | --- | --- |
|  | Univariate model | | Multivariate model | | Univariate model | | Multivariate model | |
|  | Coefficient (95% CI) | P value | Coefficient (95% CI) | P value | Coefficient (95% CI) | P value | Coefficient (95% CI) | P value |
| TAT |  |  |  |  |  |  |  |  |
| Men |  |  |  |  |  |  |  |  |
| Body fat, % |  |  |  |  |  |  |  |  |
| <25 | 0.114 (0.044-0.184) | 0.002 | -0.064 (-0.129-0.002) | 0.056 | 0.097 (0.037-0.157) | 0.002 | -0.029 (-0.145-0.088) | 0.618 |
| ≥25 | 0.059 (0.039-0.079) | <0.001 | -0.028 (-0.061-0.005) | 0.093 | 0.048 (0.017-0.078) | 0.003 | -0.035 (-0.092-0.023) | 0.226 |
| Women |  |  |  |  |  |  |  |  |
| Body fat, % |  |  |  |  |  |  |  |  |
| <35 | 0.070 (0.006-0.135) | 0.034 | -0.025 (-0.077-0.027) | 0.331 | 0.057 (-0.011-0.124) | 0.097 | -0.060 (-0.125-0.005) | 0.069 |
| ≥35 | 0.071 (0.053-0.088) | <0.001 | 0.005 (-0.028-0.039) | 0.745 | 0.052 (0.036-0.069) | <0.001 | 0.032 (-0.012-0.077) | 0.151 |
| SAT |  |  |  |  |  |  |  |  |
| Men |  |  |  |  |  |  |  |  |
| Body fat, % |  |  |  |  |  |  |  |  |
| <25 | 0.174 (0.075-0.272) | 0.001 | -0.044 (-0.151-0.063) | 0.410 | 0.144 (0.062-0.225) | 0.001 | -0.037 (-0.191-0.117) | 0.628 |
| ≥25 | 0.094 (0.066-0.122) | <0.001 | -0.012 (-0.063-0.038) | 0.616 | 0.076 (0.034-0.118) | 0.001 | -0.019 (-0.087-0.05) | 0.583 |
| Women |  |  |  |  |  |  |  |  |
| Body fat, % |  |  |  |  |  |  |  |  |
| <35 | 0.103 (0.021-0.186) | 0.016 | -0.011 (-0.074-0.051) | 0.710 | 0.085 (-0.002-0.173) | 0.056 | -0.067 (-0.162-0.028) | 0.161 |
| ≥35 | 0.099 (0.079-0.119) | <0.001 | 0.014 (-0.031-0.059) | 0.533 | 0.077 (0.055-0.098) | <0.001 | 0.046 (-0.007-0.098) | 0.087 |
| VAT |  |  |  |  |  |  |  |  |
| Men |  |  |  |  |  |  |  |  |
| Body fat, % |  |  |  |  |  |  |  |  |
| <25 | 0.162 (0.000-0.324) | 0.049 | -0.209 (-0.373--0.044) | 0.015 | 0.156 (-0.009-0.321) | 0.063 | -0.047 (-0.262-0.169) | 0.663 |
| ≥25 | 0.055 (-0.015-0.124) | 0.117 | -0.072 (-0.163-0.018) | 0.111 | 0.046 (-0.046-0.138) | 0.315 | -0.083 (-0.223-0.057) | 0.234 |
| Women |  |  |  |  |  |  |  |  |
| Body fat, % |  |  |  |  |  |  |  |  |
| <35 | 0.112 (-0.085-0.309) | 0.256 | -0.173 (-0.350-0.004) | 0.055 | 0.073 (-0.149-0.296) | 0.505 | -0.195 (-0.341--0.050) | 0.010 |
| ≥35 | 0.090 (0.011-0.170) | 0.027 | -0.015 (-0.077-0.046) | 0.619 | 0.050 (-0.018-0.118) | 0.145 | -0.001 (-0.083-0.080) | 0.972 |

Linear regression models were used to estimate the regression coefficients and 95% confidence interval (CI) for the association of fat tissue with BMD. Multivariate model was adjusted for age, race, education, family income-to-poverty ratio, smoking and drinking status, leisure activity, body mass index, calcium supplement use, vitamin D supplement use, and diabetes, and for women, hormone replacement therapy, and menopause status. **Abbreviations:** BMD bone mineral density, SAT subcutaneous adipose tissue, TAT total adipose tissue, VAT visceral adipose tissue.
